# Cultural engagement and cognitive reserve: museum attendance and dementia incidence over a 10-year period

**DOI:** 10.1192/bjp.2018.129

**Published:** 2018-11

**Authors:** Daisy Fancourt, Andrew Steptoe, Dorina Cadar

**Affiliations:** 1 Wellcome Research Fellow, Department of Behavioural Science and Health, University College London, UK; 2 Professor, Department of Behavioural Science and Health, University College London, UK; 3 Research Associate, Department of Behavioural Science and Health, University College London, UK

**Keywords:** Dementia, cultural engagement, museums, social engagement, cognitive reserve

## Abstract

Theories of cognitive reserve, disuse syndrome and stress have suggested that activities that are mentally engaging, enjoyable and socially interactive could be protective against the development of dementia. Using data from the English Longitudinal Study of Ageing, this study shows that for adults aged 50 and older visiting museums every few months or more was associated with a lower incidence rate of dementia over a 10-year follow-up period compared with less-frequent visiting. This association was independent of demographics, socioeconomic status, health-related variables including sensory impairment, depression, vascular conditions and other forms of community engagement. Visiting museums may be a promising psychosocial activity to support the prevention of dementia.

**Declaration of interest:**

None.

Neuropsychological and psychiatric studies have presented several theories on how dementia can be prevented or delayed. Theories of cognitive reserve suggest that complex and stimulating experiences can enhance neuronal structure and brain function, protecting against neurodegeneration.^1^ ‘Disuse syndrome’ proposes that a lack of cognitive stimulation in everyday life leads to faster deterioration in cognitive function.^2^ Similarly, studies on arousal and hedonic tone have shown that cognitive flexibility can be improved through positive affect,^3^ demonstrating a bidirectional relationship between well-being and cognitive function.^4^ Moreover, social engagement has been found to support resilience, which has a neuroprotective effect.^5^ Consequently, activities that are mentally engaging, enjoyable, stress-reducing and socially interactive could be protective against the development of dementia, and in light of this, there has been a call for the identification of more affordable multimodal public health interventions to decrease the risk of dementia for individuals.^6^

A multimodal activity that combines a number of protective factors (including intellectual stimulation, light physical activity, positive affect, relaxation and social engagement through interaction with staff, fellow visitors or friends) is visiting museums, art galleries and exhibitions (hereafter referred to as ‘museums’).^7^^–^^9^ Previous studies have incorporated ‘visiting museums’ within definitions of leisure activities in dementia research,^10^ or looked at the broader impact of attendance on people with dementia, finding benefits for cognitive performance, well-being, quality of life, self-esteem and social support.^11^ However, this is the first study (to the authors’ knowledge) to explore whether visiting museums among adults aged 50 and older is associated with a lower incidence rate of dementia over a 10-year period. The study has ethical approval from the National Research Ethics Service.

## Method

### Participants

We analysed data from the English Longitudinal Study of Ageing (ELSA): a large, nationally representative, multidisciplinary cohort study of the English population aged 50 and older.^12^ We included participants who were core members in the study, who were free of dementia at baseline (wave 2) and who provided data across all variables of interest at 10-year follow-up (wave 7) (*n* = 3911).

### Measures

#### Cultural engagement

Visiting museums, art galleries and exhibitions was measured using a self-report scale asking about frequency of engagement (‘never’, ‘less than once a year’, ‘about once or twice a year’, ‘every few months’, ‘about once a month’ or ‘twice a month or more’). We collapsed the final three categories together to provide an overall four-point scale.

#### Dementia

Dementia occurrence was determined at each wave by using an algorithm based on a combination of self- or informant-reported physician diagnosis of dementia or Alzheimer's disease, or a score above the threshold of 3.38 on the 16-question Informant Questionnaire on Cognitive Decline in the Elderly (IQCODE). Memory complaints captured by IQCODE have previously been validated as a predictor of dementia.^13^

#### Covariates

Based on previous analyses, we identified a number of potentially confounding variables including demographic covariates (gender, age, marital status, educational attainment, employment, wealth and previous occupational classification), health-related covariates (eyesight, hearing, depression and existing cardiovascular health conditions) and community engagement (including membership of social clubs, arts or music groups, charities, church groups, volunteer networks, political or union groups, neighbourhood groups, environmental groups or sports clubs).

#### Statistical analyses

Incidence rates (IR) of dementia were computed per 1000 person-years in relation to museum visits frequency. We used Poisson regression analyses to calculate the incidence rate ratio (IRR) of dementia incidence and 95% CIs. Model 1 was unadjusted, model 2 adjusted for demographics, model 3 additionally adjusted for health-related factors, and model 4 was additionally adjusted for other indicators of community engagement. All analyses were weighted using baseline cross-sectional weights derived from ELSA to ensure the sample was representative of the English population. We applied three types of sensitivity analyses. To explore in more detail how age might affect the relationship observed, sensitivity analyses first included age as an interaction term, and then split participants into those above (51.6%) and below (48.4%) the age of 65. To confirm that analyses were not biased by the inclusion of participants already experiencing preclinical symptoms of dementia, we excluded all participants who developed dementia in the 2 years following baseline and re-ran analyses. Finally, in order to account for non-response on cultural participation (15.4%), we imputed missing data on cultural participation using chained equations, which included all health-related variables in the prediction model to generate 100 imputed data-sets (each had a final *n* = 4607). The missing-at-random assumption was strengthened by the fact that some of the same variables used to predict cultural engagement are also known to predict non-response in ELSA (including age, education and wealth).^12^ Sensitivity analyses are shown in supplementary Tables 1–3 (available at https://doi.org/10.1192/bjp.2018.129). Analyses were carried out using Stata SE Version 14.1.

## Results

Our sample included 3911 adults (45% male, 55% female; mean age 63.8 years, s.d. = 8.3), of whom 246 (6.3%) developed dementia during the 10-year study period. A total of 32.7% reported never attending an art gallery or museum, 26.8% reported attending less than once a year, 21.6% reported attending once or twice a year, and 18.9% reported attending every few months or more. The overall incidence rate was 5.42 (95% CI 4.78–6.17) per 1000 person-years. As anticipated, there was an above-average incidence rate for those who never visited (IR = 9.47, 95% CI 8.02–11.25), a slightly below-average rate for those who visited rarely (less than once a year: IR = 3.96, 95% CI 3.03–5.29; once or twice a year: IR = 3.73, 95% CI 2.70–5.30) and the lowest rate for those visited every few months or more (IR = 2.15, 95% CI 1.41–3.48).

The multivariable analyses are summarised in Table 1. Attending museums at any level of frequency was associated with a lower dementia incidence in unadjusted analyses. However, demographic variables accounted for much of this association, such that after this adjustment, the association only held for museum attendance every few months or more (IRR = 0.47, 95% CI 0.29–0.75). Further accounting for health-related variables and community engagement did not account for much more of the remaining association (IRR = 0.50, 95% CI 0.31–0.81 and IRR = 0.51, 95% CI 0.32–0.83, respectively).

**Table 1:** Associations between visiting art galleries and museums and dementia incidence

	Incidence rate ratio (s.e.) 95% CI	*P*
Model 1		
Never	Reference	
Less than once a year	**0.43** (**0.07) 0.31–0.59**	**<0.001**
Once or twice a year	**0.40** (**0.07) 0.28–0.58**	**<0.001**
Every few months	**0.23** (**0.06) 0.15–0.37**	**<0.001**
Model 2		
Never	Reference	
Less than once a year	0.83 (0.13) 0.61–1.12	0.23
Once or twice a year	**0.69 (0.13) 0.482–1.00**	**0.05**
Every few months	**0.47 (0.11) 0.29–0.75**	**0.002**
Model 3		
Never	Reference	
Less than once a year	0.89 (0.14) 0.65–1.21	0.45
Once or twice a year	0.74 (0.14) 0.51–1.07	0.11
Every few months	**0.50 (0.12) 0.31–0.81**	**0.005**
Model 4		
Never	Reference	
Less than once a year	0.92 (0.15) 0.67–1.26	0.61
Once or twice a year	0.76 (0.14) 0.53–1.09	0.14
Every few months	**0.51 (0.13) 0.32–0.83**	**0.006**

Results in bold are significant. Model 1: unadjusted. Model 2: adjusted for gender, age, marital status, educational attainment, employment, wealth and occupational classification. Model 3: additionally adjusted for eyesight, hearing, depression and existing cardiovascular health conditions. Model 4: additionally adjusted for community engagement.

Sensitivity analyses showed that age was a partial moderating factor (for every few months or more, IRR = 1.06, 95% CI 1.02–1.12) but examinations of the data for those above and below 65 showed similar patterns of association (age 50–64: IRR = 0.12, 95% CI 0.02–0.83; age ≥65: IRR = 0.64, 95% CI 0.39–1.03). Results were unaffected by omitting participants who might have been experiencing preclinical symptoms and developed dementia in the 2 years of follow-up (for every few months or more, IRR = 0.49, 95% CI 0.29–0.82) or by using multiple imputation to account for the 15.4% who were missing baseline data on cultural participation (*n* = 4607; for every few months or more, IRR = 0.58, 95% CI 0.37–0.92). In order to isolate visiting museums from other types of cognitive activities, we also ran correlational analyses with activities such as going to the theatre, concerts or opera and reading newspapers. Correlations were low, suggesting visiting museums is distinct from other cognitive activities, and including these further variables in the model did not affect the significance of results (but did reduce sample size because of missing data; data available from the authors on request).

## Discussion

This study showed for the first time that among people who visit museums every few months or more, there is a lower incidence rate of dementia over a 10-year follow-up period. Although much of the association was explained by demographic and socioeconomic variables, it is notable that the relationship for more frequent engagement was maintained even when controlling for these confounders. The inclusion of further confounders such as health-related variables and other forms of community engagement had very little effect on results, suggesting that the association between visiting museums and dementia onset is independent of factors such as sensory impairment, depression and vascular conditions and separate from multiple further types of community social engagement. The combined neural and sensory stimulation and cognitive engagement provided by museums, make attendance a potential cultural intervention for increasing or maintaining cognitive reserve.^14^ Visiting museums is also a light physical activity so could reduce the negative effects of sedentary behaviours. Further, visiting museums can be seen as a specific type of social engagement: visiting can reduce perceived isolation by encouraging people to leave their homes, it is an activity that is frequently a focal point for meeting family/friends, and even if people attend alone, there is casual social contact with museum staff and/or other visitors. These findings therefore build on a previous study which examined social engagement as a determinant for cognitive reserve, demonstrating associations with better global cognition in early Parkinson's disease and lower risk of dementia incidence.^15^

This study has a number of strengths: through the extensive biennial monitoring of an extensive, nationally representative cohort study, we were able to measure 10-year dementia incidence. We considered non-response and attrition through the use of inverse probability weighting and multiple imputation. The comprehensive inclusion of covariates in ELSA meant that we could control for identified confounding variables. However, this study is observational rather than experimental and it is still possible that unidentified confounding might have affected these findings, or that selected variables might incompletely control for the constructs they represent. In particular, it is recognised that cultural engagement has a social gradient. However, our analyses included variables on education, wealth and socioeconomic classification, with results maintained independent of these factors, suggesting that going to museums is more than just a proxy for broader social factors already known to be protective against dementia. Further, our ascertainment of dementia incidence may have been underestimated because of recognised diagnostic challenges to date. Finally, it is recognised that subtle cognitive and behavioural changes may precede dementia diagnoses by more than a decade. Although our sensitivity analyses excluding participants who developed dementia in the 2 years following baseline showed no substantial changes to results, as more data become available it will be relevant to test associations between cultural engagement and dementia across longer timespans.

Further studies may wish to extend these findings by exploring more the cognitive mechanisms underlying the results reported here, considering the relationship between museum visits and incidence rates for more specific types of dementia as well as focusing in particular on subgroups at high risk, and exploring potential protective effects of other types of multimodal cultural activities. In conclusion, this novel analysis demonstrates that cultural participation through museum attendance could provide opportunities for interventions in older adults as a way of supporting engaged lifestyles to prevent dementia. Given museums number around 40 000 in Europe, the USA and Canada and reach diverse geographical populations and demographic groups, their potential could be explored further as sites for public health interventions.^9^

## Supplementary material

For supplementary material accompanying this paper visit https://doi.org/10.1192/bjp.2018.129. click here to view supplementary material
