# Supplementary material for: Cultural engagement and cognitive reserve: museum attendance and dementia incidence over a 10-year period
Source: Br J Psychiatry. 2018 Nov;213(5):661–3. doi: 10.1192/bjp.2018.129 (PMC6429239; doi:10.1192/bjp.2018.129)
Supplement: Supplementary file 1 [file S0007125018001290sup001.docx]

**SUPPLEMENTARY MATERIAL: SENSITIVITY ANALYSES**

**Table S1: Associations between visiting art galleries and museums and dementia incidence by age**

|  | Age 50-64 (n=2,269) | | | Age 65+ (n=1,642) | | |
| --- | --- | --- | --- | --- | --- | --- |
|  | IRR ± SE | 95% CI | p-value | IRR ± SE | 95% CI | p-value |
| MODEL 1 |  |  |  |  |  |  |
| Never | REF |  |  |  |  |  |
| Less than once a year | 0.92 ± 0.40 | 0.40-2.15 | .85 | 0.51 ± 0.09 | 0.37-0.71 | <.001 |
| Once or twice a year | 0.75 ± 0.40 | 0.27-2.13 | .59 | 0.45 ± 0.09 | 0.31-0.65 | <.001 |
| Every few months | 0.10 ± 0.10 | 0.01-0.74 | .025 | 0.33 ± 0.08 | 0.21-0.52 | <.001 |
| MODEL 2 |  |  |  |  |  |  |
| Never | REF |  |  |  |  |  |
| Less than once a year | 1.28 ± 0.54 | 0.56-2.91 | .56 | 0.75 ± 0.13 | 0.54-1.05 | .10 |
| Once or twice a year | 1.10 ± 0.66 | 0.34-3.54 | .87 | 0.64 ± 0.12 | 0.44-0.93 | .02 |
| Every few months | 0.13 ± 0.13 | 0.02-0.91 | .040 | 0.55 ± 0.13 | 0.34-0.88 | .014 |
| MODEL 3 |  |  |  |  |  |  |
| Never | REF |  |  |  |  |  |
| Less than once a year | 1.30 ± 0.55 | 0.56-3.00 | .54 | 0.82 ± 0.14 | 0.58-1.15 | .25 |
| Once or twice a year | 1.13 ± 0.68 | 0.34-3.70 | .84 | 0.69 ± 0.13 | 0.47-1.00 | .05 |
| Every few months | 0.13 ± 0.13 | 0.02-0.92 | .040 | 0.59 ± 0.14 | 0.36-0.95 | .031 |
| MODEL 4 |  |  |  |  |  |  |
| Never | REF |  |  |  |  |  |
| Less than once a year | 1.26 ± 0.54 | 0.54-2.91 | .60 | 0.86 ± 0.15 | 0.61-1.22 | .41 |
| Once or twice a year | 1.04 ± 0.62 | 0.32-3.37 | .94 | 0.72 ± 0.14 | 0.49-1.05 | .09 |
| Every few months | 0.12 ± 0.12 | 0.02-0.83 | .032 | 0.64 ± 0.16 | 0.39-1.03 | .067 |

Model 1: unadjusted. Model 2: adjusted for sex, age, marital status, educational attainment, employment, wealth and occupational classification. Model 3: additionally adjusted for eyesight, hearing, depression and existing cardiovascular health conditions. Model 4: additionally adjusted for community engagement.

**Table S2: Associations between visiting art galleries and museums and dementia incidence omitting participants who developed dementia in the two years following baseline**

|  | n=3,884 | | |
| --- | --- | --- | --- |
|  | IRR ± SE | 95% CI | p-value |
| MODEL 1 |  |  |  |
| Never | REF |  |  |
| Less than once a year | 0.48 ± 0.08 | 0.35-0.67 | <.001 |
| Once or twice a year | 0.41 ± 0.08 | 0.28-0.61 | <.001 |
| Every few months | 0.23 ± 0.06 | 0.14-0.38 | <.001 |
| MODEL 2 |  |  |  |
| Never | REF |  |  |
| Less than once a year | 0.89 ± 0.14 | 0.65-1.22 | .48 |
| Once or twice a year | 0.68 ± 0.14 | 0.46-1.01 | .057 |
| Every few months | 0.45 ± 0.12 | 0.27-0.76 | .003 |
| MODEL 3 |  |  |  |
| Never | REF |  |  |
| Less than once a year | 0.93 ± 0.15 | 0.68-1.28 | .65 |
| Once or twice a year | 0.71 ± 0.14 | 0.48-1.05 | .09 |
| Every few months | 0.48 ± 0.13 | 0.29-0.80 | .005 |
| MODEL 4 |  |  |  |
| Never | REF |  |  |
| Less than once a year | 0.95 ± 0.15 | 0.69-1.30 | .74 |
| Once or twice a year | 0.72 ± 0.15 | 0.49-1.07 | .11 |
| Every few months | 0.49 ± 0.13 | 0.29-0.83 | .007 |

Model 1: unadjusted. Model 2: adjusted for sex, age, marital status, educational attainment, employment,wealth occupational classification. Model 3: additionally adjusted for eyesight, hearing, depression and existing cardiovascular health conditions. Model 4: additionally adjusted for community engagement.

**Table S3: Associations between visiting art galleries and museums and dementia incidence imputing missing data on cultural attendance**

|  | N=4,607 | | |
| --- | --- | --- | --- |
|  | IRR ± SE | 95% CI | p-value |
| MODEL 1 |  |  |  |
| Never | REF |  |  |
| Less than once a year | 0.47 ± 0.07 | 0.35-0.63 | <.001 |
| Once or twice a year | 0.40 ± 0.07 | 0.28-0.57 | <.001 |
| Every few months | 0.26 ± 0.06 | 0.16-0.40 | <.001 |
| MODEL 2 |  |  |  |
| Never | REF |  |  |
| Less than once a year | 0.88 ± 0.13 | 0.67-1.17 | .38 |
| Once or twice a year | 0.73 ± 0.13 | 0.52-1.03 | .071 |
| Every few months | 0.55 ± 0.12 | 0.35-0.85 | .008 |
| MODEL 3 |  |  |  |
| Never | REF |  |  |
| Less than once a year | 0.93 ± 0.13 | 0.70-1.23 | .59 |
| Once or twice a year | 0.76 ± 0.13 | 0.54-1.07 | .11 |
| Every few months | 0.57 ± 0.13 | 0.37-0.90 | .015 |
| MODEL 4 |  |  |  |
| Never | REF |  |  |
| Less than once a year | 0.94 ± 0.14 | 0.71-1.25 | .67 |
| Once or twice a year | 0.77 ± 0.13 | 0.55-1.09 | .14 |
| Every few months | 0.58 ± 0.13 | 0.37-0.92 | .02 |

Model 1: unadjusted. Model 2: adjusted for sex, age, marital status, educational attainment, employment, wealthand occupational classification. Model 3: additionally adjusted for eyesight, hearing, depression and existing cardiovascular health conditions. Model 4: additionally adjusted for community engagement.
